# A Survey of Attitudes, Anxiety Status, and Protective Behaviors of the University Students During the COVID-19 Outbreak in Turkey

**DOI:** 10.3389/fpsyt.2020.00695

**Published:** 2020-07-15

**Authors:** Gulsum Akdeniz, Mariam Kavakci, Muharrem Gozugok, Semiha Yalcinkaya, Alper Kucukay, Bilal Sahutogullari

**Affiliations:** ^1^ Department of Neuroscience, Ankara Yildirim Beyazit University, Ankara, Turkey; ^2^ Electroneurophysiology Lab, Department of Biophysics, and Yenimahalle Training and Research Hospital, Ankara Yildirim Beyazit University, Ankara, Turkey; ^3^ Department of Translational Medicine, Ankara Yildirim Beyazit University, Ankara, Turkey

**Keywords:** COVID-19, coronavirus disease, behavioral, outbreak, anxiety, survey

## Abstract

A new coronavirus disease began on 31 December 2019 in Wuhan/China and has caused a global outbreak in only a few months resulting in millions being infected. In conjunction with its’ physical side effects, this outbreak also has a tremendous impact on psychology health. This study aims to assess the spread and frequency of protective behaviors, emotional and anxiety status among the Turkish population using a rapid survey during the COVID-19 outbreak. An online questionnaire was administered to 3,040 respondents between the ages of 18–30. This cross-sectional study was conducted from Apr 2 to Apr 8, 2020. While questions related to the outbreak were created by members of our neuroscience department, the Turkish version of the Abbreviated Beck Anxiety Inventory was included in our survey to measure anxiety status. Pearson correlation coefficient was used for statistical analysis. We found that 90% of respondents report washing hands more frequently since the outbreak while %50 wear protective gloves. Respondents were more fearful of their relatives catching the coronavirus disease than they were of themselves catching it. In response to the question, “What are your emotions about the coronavirus?”, 38% responded with “worried”. There was a significant correlation between anxiety status and consumption information from the media about COVID-19. Individual early protection behaviors might slow transmission of the outbreak. Our results showed that the behavior of the participants has changed in predictable ways during the COVID-19 outbreak. Understanding how emotional responses such as fear and anxiety status vary and the specific factors that mediate it may help with the design of outbreak control strategies.

## Introductıon

Novel coronavirus disease (COVID-19) began in Wuhan, China in December 2019 and has spread worldwide since then. This new coronavirus disease turned into an outbreak reaching around the world in as little as three months showing the serious threat of this outbreak. The first patient with coronavirus disease was identified in Turkey on March 10, 2020 (The Republic of Turkey, Ministry of Health, 02/04/2020).

People display awareness of protective behaviors against diseases and develop health-protective attitudes during a health crisis, such as an outbreak. Timely and accurate information plays a critical role in controlling the spread of illness and managing fear and uncertainty during an outbreak. Furthermore, society’s perception of risk and anxiety of being ill have an impact on prevention behaviors and measures to be taken. Knowing what to do helps people feel safer and enhances the belief that they can take meaningful steps to protect themselves (1). In outbreaks, anxiety is one of the psychological problems that can be seen in humans because pandemics can be seen as events that can raise concerns. The use of a face mask (2) and protective measures during the workplace are protective factors for mental health (3). Anxiety and related disorders (such as posttraumatic stress disorder, and obsessive-compulsive disorder) can be seen due to stressful life events, and they are prevalent, debilitating, and costly (4, 5). The outbreak of COVID-19 has been reported to cause mental health problems among the people in China (6), Japan (7), and Wuhan (8).

Due to the sudden nature of the outbreak and the infectious power of the coronavirus, people may show psychological and stress-related reactions. Some prohibitions and precautions were taken against the coronavirus disease outbreak such as social isolation, quarantine, travel restrictions, contact avoidance. These measures affect people’s social life, emotional status, and psychological well-being. It is necessary to investigate and understand the publics’ mental states during this tumultuous time (6). Accordingly, psychological and behavioural measurement and evaluation are essential. Psychological tests contribute to the identification of certain disorders, monitoring of disease, and make predictions in a way that reflects the variability in human behaviors (9). Furthermore, psychological tests such as web-based surveys offer a rapid and efficient method of identifying problems, planning and monitoring a course of treatment, and assessing the outcomes of interventions (10). Particularly in the severe COVID-19 pandemic, the data obtained through these methods provide information about people’s attitudes, emotions, and behaviors while providing a contemporary perspective to researchers. However, what type of psychological disorders are prevalent and how they distribute among the population are not know. Therefore, a rapid assessment of outbreak-associated psychological disorders for the public is needed (11). So, the current study aims to determine the prevalence and distribution of anxiety and emotional status and protective behaviors among the young Turkish population and examine their associations with media exposure with a rapid assessment during the COVID-19 outbreak.

## Materıals and Methods

### Participants and Design

This cross-sectional study was conducted online over a span of seven days from April 2 to April 8, 2020. Participants were 3040 university students living in Turkey. Google documents were used as a platform to design online surveys that were automatically hosted *via* a unique URL. The survey was created by the Department of Neuroscience at Ankara Yildirim Beyazit University. Respondents were asked about about (i) demographic and epidemiological information, (ii) protective behaviors to prevent catching the coronavirus, (iii) different emotions and thoughts caused by the COVID-19 outbreak, (iv) anxiety status during the COVID-19 outbreak, and (v) exposure to COVID-19 Outbreak on TV. Respondents had to answer a yes-no question to confirm their willingness to participate voluntarily. After confirmation of the question, the participant was directed to complete the self-report survey. Respondents were found from internet social media tools such as Facebook and Twitter.

### Measures

Respondents gave free-text responses to questions about their name-surname, current age, and city.

### Protective Behaviors in Response to COVID-19

To measure the response of epidemiologically relevant behavior to information on the coronavirus disease outbreak, we asked seven yes/no questions about precaution actions taken by the respondents. In the survey, we asked: “washing hands more often with soap for 20 seconds”, “wearing protective gloves”, “wearing a mask”, “avoiding contact with hands, face, and eyes”, “washing clothes at a minimum of 60 degrees”, “personal and social isolation”, and “frequent ventilation of the room”. All of these actions are recommended as protective measures by doctors.

### Emotional and Anxiety Status With COVID-19 Outbreak

An important epidemiological question is how people’s affective states and anxiety have undertaken change with progression of the outbreak. To measure this, we asked participants two critical questions:

“How scared are you of catching the coronavirus disease (COVID-19)?”

“How scared are you that a relative will catch the coronavirus disease (COVID-19)?”

These questions were asked using a five-point ordinal scale with anchors at all points: “never”, “somewhat”, “moderate”, “very”, and “extremely”. The two questions were compared to each other for the frequency distribution of perceived risk and fear of the new coronavirus disease outbreak.

To assess emotional status in the survey, we asked: “What are your emotions about the coronavirus?”. The respondents were asked to choose from five different emotions. The choices were: “afraid”, “sad”, “worried”, “indifferent”, and “temporary”.

Respondents were also asked about anxiety status during the coronavirus disease outbreak. We used eight-items from the Turkish version of Abbreviated Beck Anxiety Inventory: “fear of death”, “scared”, “difficulty in breathing”, “fear of losing control”, “feeling of choking”, “nervous”, “terrified or afraid”, and “fear of the worst happening” (12, 13). Additionally, we added the following anxiety statuses: “Fear of losing your relative”, “sad”, “future anxiety”. A self-report measure of anxiety severity experienced in the last 15 days was also included. These statements were asked using a five-point ordinal scale with anchors at all points.: “never”, “somewhat”, “moderate”, “serious”, and “very serious”.

For our analysis of participants’ responses to the threat of the coronavirus disease (COVID-19), we used a variable called “survey day”. April 2nd represents the first day of the survey and April 8th represents the last day. This survey was joined one time by each participant. We investigated the change in respondents’ protective behavior status and emotional status for each day in the survey.

### Exposure to COVID-19 Outbreak on TV

Media exposure was evaluated by asking how often respondents were exposed to news and information about COVID-19 on TV over the past fifteen days. Response options were “never”, “1–3 hours”, “3–5 hours”, “5–7 hours”, and “7 or more hours”. The correlation between protection behavior, sleep status, emotional status and information about COVID-19 from the TV was investigated.

### Statistical Analysis

Statistical analyses were performed with the Statistical Package for the Social Sciences (SPSS 22.0, SPSS Inc., Chicago, IL) software. The Pearson correlation coefficient (PCC) was used to evaluate a possible correlation between information about COVID-19 on TV and protection behavior after determining the normal distribution of data. To assess the normality of a set of data, researchers usually report the Skewness and Kurtosis of such data. Normality is tested according to the common rule-of-thumb, which is to run descriptive statistics to determine both Skewness and Kurtosis. PCC was used to assess the relationship between information about COVID-19 on the TV and emotional status. PCC was also used to analyze the relationship between information about COVID-19 on TV and sleep status. Statistical tests were carried out with a level of significance at *p*=0.05.

## Results

**Figure 1:**
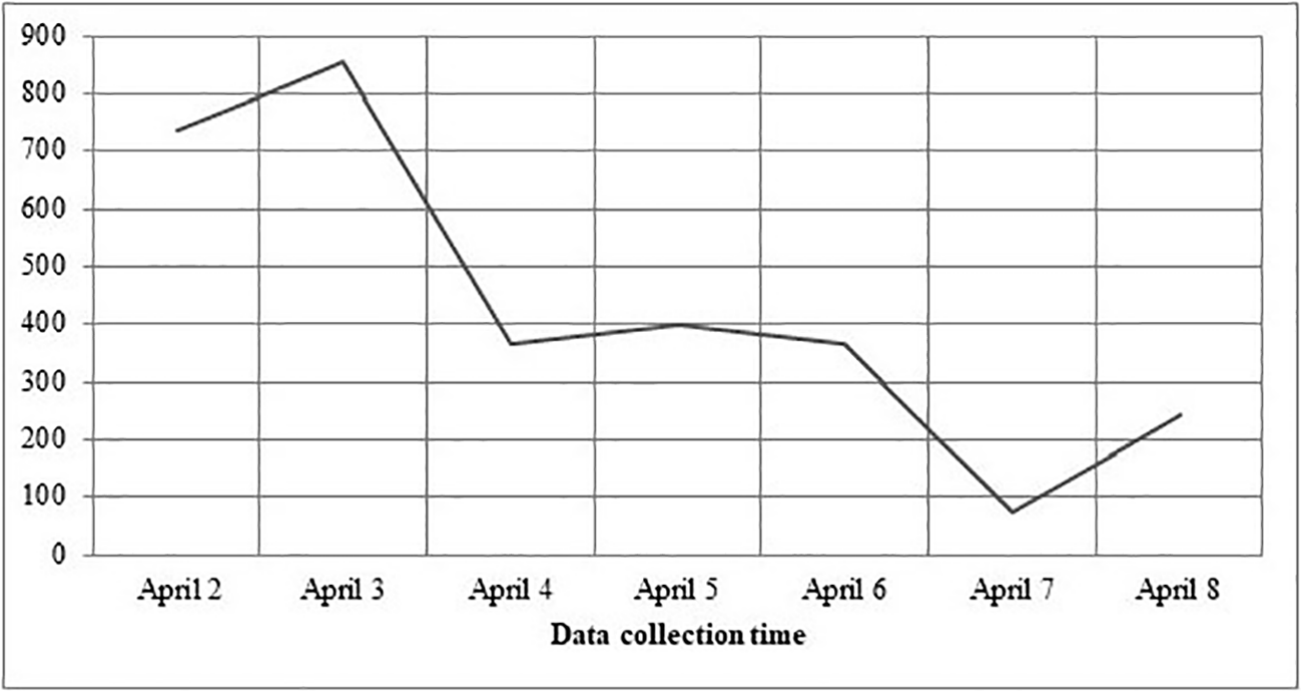
Presents the daily distribution respondents to the survey.

A total of 3,040 Turkish university students (77,5% female, 22,5% male) ages 18–30 (20,7 ± 2,2) filled out the online survey named “Web-based Behavioral Measurement Related to COVID-19*”*. **Figure 1** shows the distribution of the survey date and the number of respondents. **Table 1** displays the epidemiological and demographic data of the respondents.

**Table 1:** Demographic data of the study population.

Variable	Respondents
Total	3040
**Gender**	
Female	2355 (77.5%)
Male	685 (22.5%)
**Age (years)**	
18-20	1370 (45.1%)
21-23	1321 (43.5%)
24-30	349 (11.4%)
**Smoking Status**	
Smokers	639 (21%)
Non-smokers	2401 (79%)
**Chronic disease**	
Yes	258 (8.5%)
No	2782 (91.5%)
**Physical activity**	
Yes No	1570 (51.6%) 1470 (48.4%)

**Figure 2:**
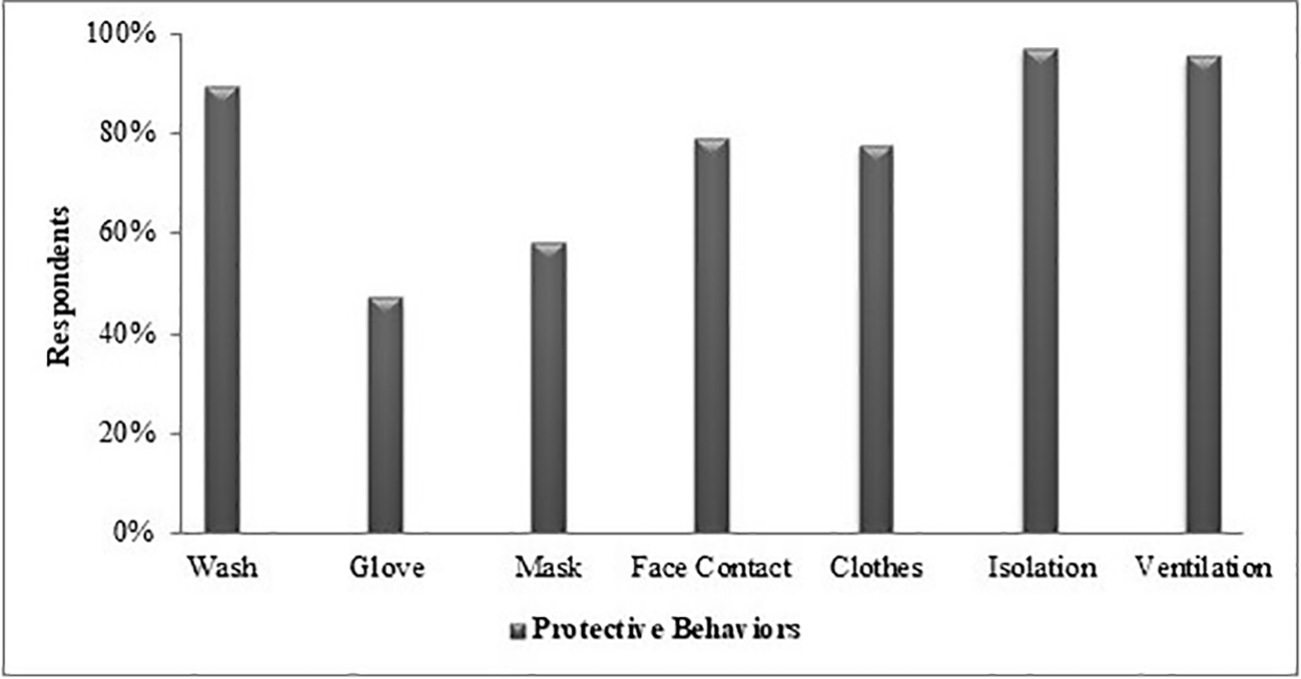
Frequency of the protection behavior undertaken by the respondents, %. Wash, washing hands frequently with soap for 20 s; glove, wear protective gloves when going out; mask, wear protective mask when going out; face contact, avoid touch with hands face and eyes; cloth, washing clothes at least 60 degrees; isolation, personal and social isolation; ventilation, frequent ventilation of the room.

**Figure 2** shows the frequency distribution of protective behavior. Respondents paid attention to hand washing (90%), social isolation (97%), and room ventilation (95%). The rate of wearing protective gloves and masks is notably lower (50%).

**Figure 3:**
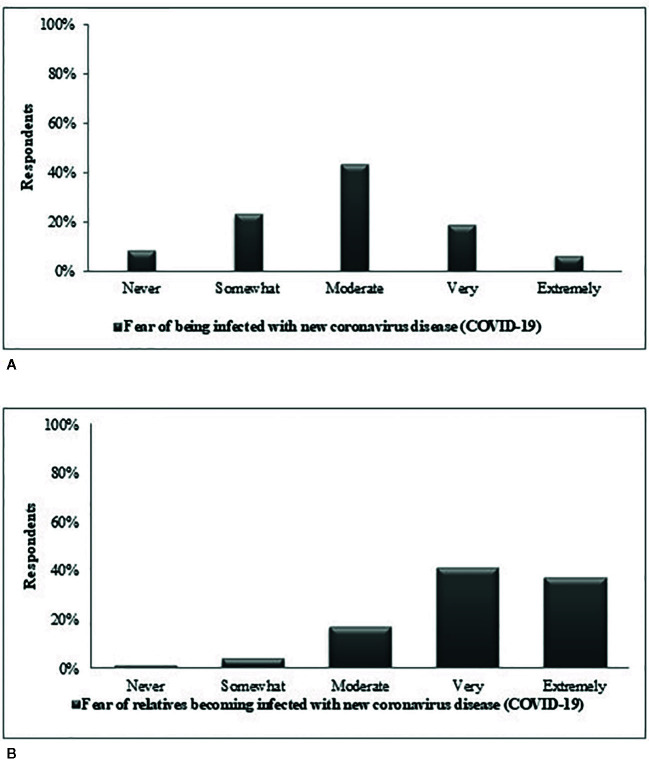
Frequency distribution of fear of relatives infected with new coronavirus disease (COVID-19) **(A)** Oneself cathing **(B)** Relative cathing the disease.

**Figures 3A, B** reveal the frequency distribution of perceived COVID-19 risk and fear for respondents and their relatives. Respondents had to moderate fear of catching constituted 44% while 9% stated that they did not have this fear. Respondents’ fear of their relatives being infected with the disease was much higher with 80% of them reporting their fear as high and extreme.

**Figure 4:**
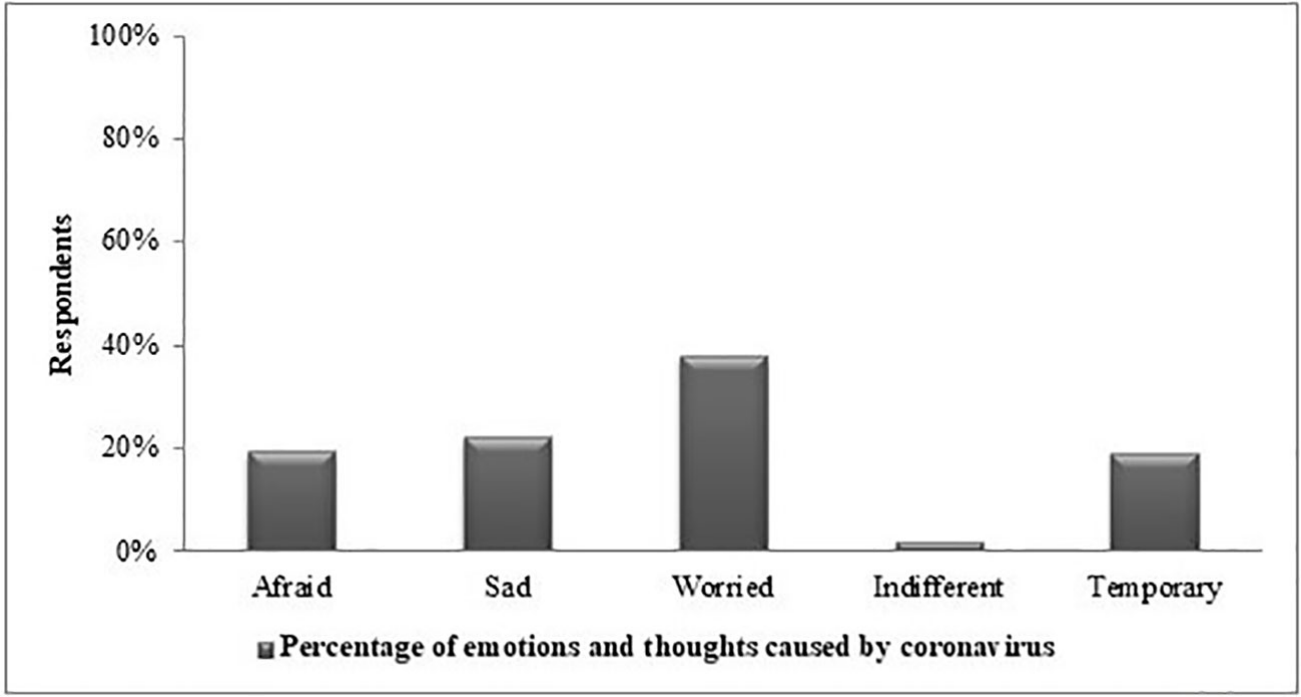
Frequency distribution of emotional status about COVID-19 by respondents.

**Figure 4** includes responses to the question “What are your emotions about the coronavirus?”. While 38% of the respondents stated that they were worried about the new coronavirus, there was a 2% portion that reported they were indifferent. In addition, 20% of the respondents’ perceived this virus as temporary.

According to Tabachnick, data may be assumed to be normal if both Skewness and Kurtosis are within a value range of ±1.5. **Tables 2** and **3** present the results of Skewness and Kurtosis analysis on each of the items that measure the constructs of our study (14). There was a significant correlation between being exposed to information about COVID-19 on TV, hand washing, and clothes (**Table 2**). However, as shown in **Table 3** no significant correlation was found between being exposed to information about COVID-19 on TV and other precautions. The correlations are summarized in **Tables 2** and **3**.

**Table 2:** Correlation between information about COVID-19 on the TV and protection behaviors.

	Wash	Mask	Gloves	Clothes	Ventilation	Face contact	Isolation
**Information**							
**PCC**	0.042*	0.014	0.005	0.039*	0.030	0.029	0.015
**p-value** **Skewness** **Kurtosis**	0.020 1.328 -0.237	0.450 0.842 -0.611	0.784 0.735 -1.371	0.031 0.933 -1.134	0.102 0.620 -1.003	0.108 0.542 -0.463	0.424 0.723 -0.691

PCC, Pearson correlation coefficent; Information, Being exposed to information about COVID-19 on TV; p < 0.05. *, statistically significant.

**Table 3:** **(A)** Correlation between sleep status and exposure to information (TV, social media); (**B)** Correlation between information about COVID-19 on the TV and emotional status.

A	Information	Watch TV	PC	MP
**Sleep status**				
**PCC**	0.017	0.005	0.066*	0.043*
**p-value** **Skewness** **Kurtosis**	0.335 0.817 -0.733	0.425 0.737 -1.464	0.000 0.821 -0.337	0.018 0.309 -1.020
**B**	**Fear**	**Fear of death**	**Fear of losing your relative**	**Future anxiety**
**Information**				
**PCC**	0.157*	0.179*	0.107*	0.146*
**p-value** **Skewness** **Kurtosis**	0.0 0.517 -0.406	0.0 0.716 -0.176	0.0 0.101 -0.138	0.0 0.128 -1.005

PCC, Pearson correlation coefficent; Information, Being exposed to information about COVID-19 on TV; PC, Spending time on the computer; MP, Spending time on a mobile phone; p < 0.05. *, statistically significant.

**Figure 5:**
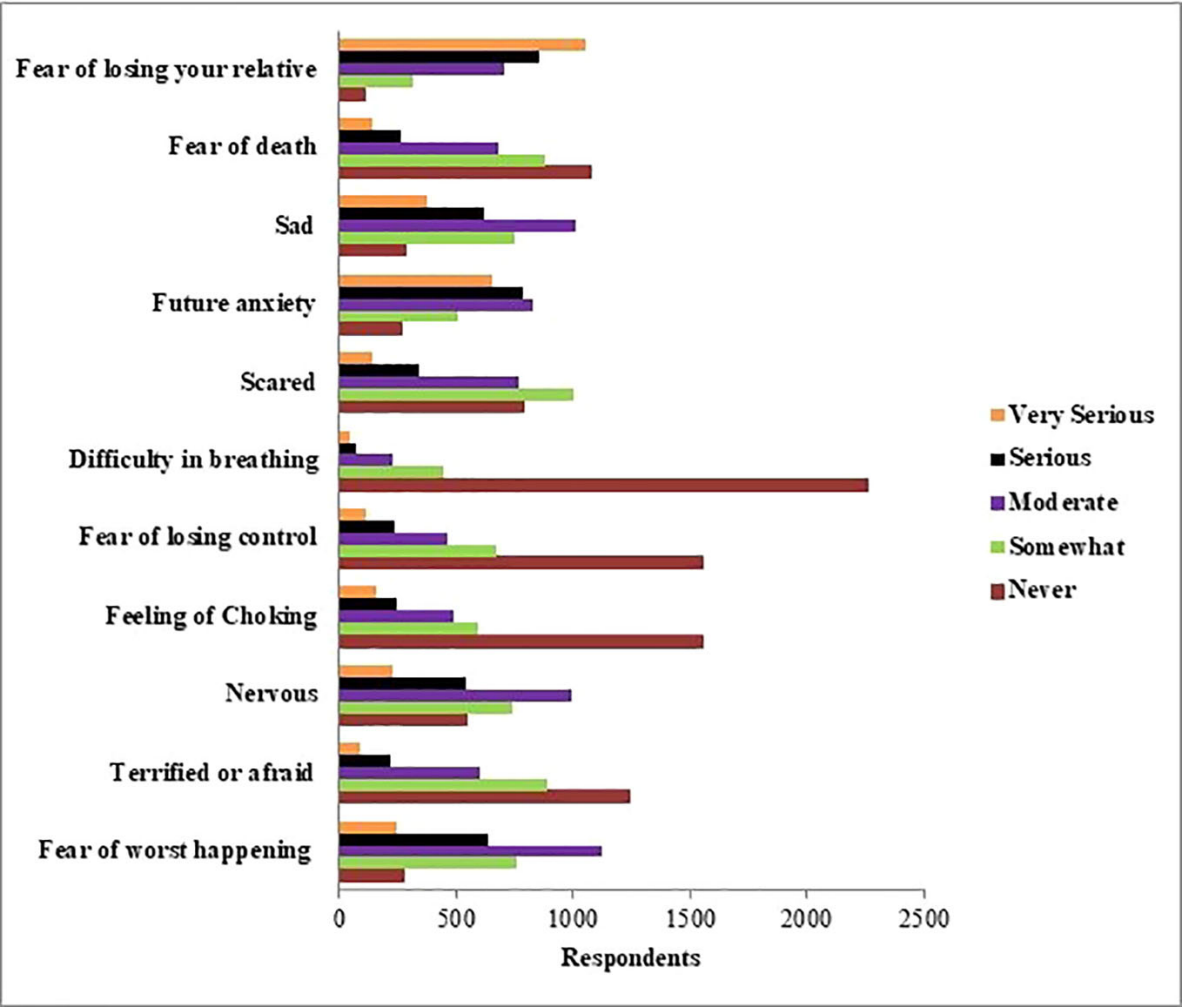
Distribution of anxiety status during the COVID-19 outbreak.

In **Figure 5**, we show the distribution of anxiety status during the COVID-19 outbreak. “Serious” and “very serious” options were high among the responses given to “future anxiety” and “fear of losing relatives”. In addition, the “never” option was high in response to the questions about “difficulty in breathing” and “feeling of choking”.

**Figure 6:**
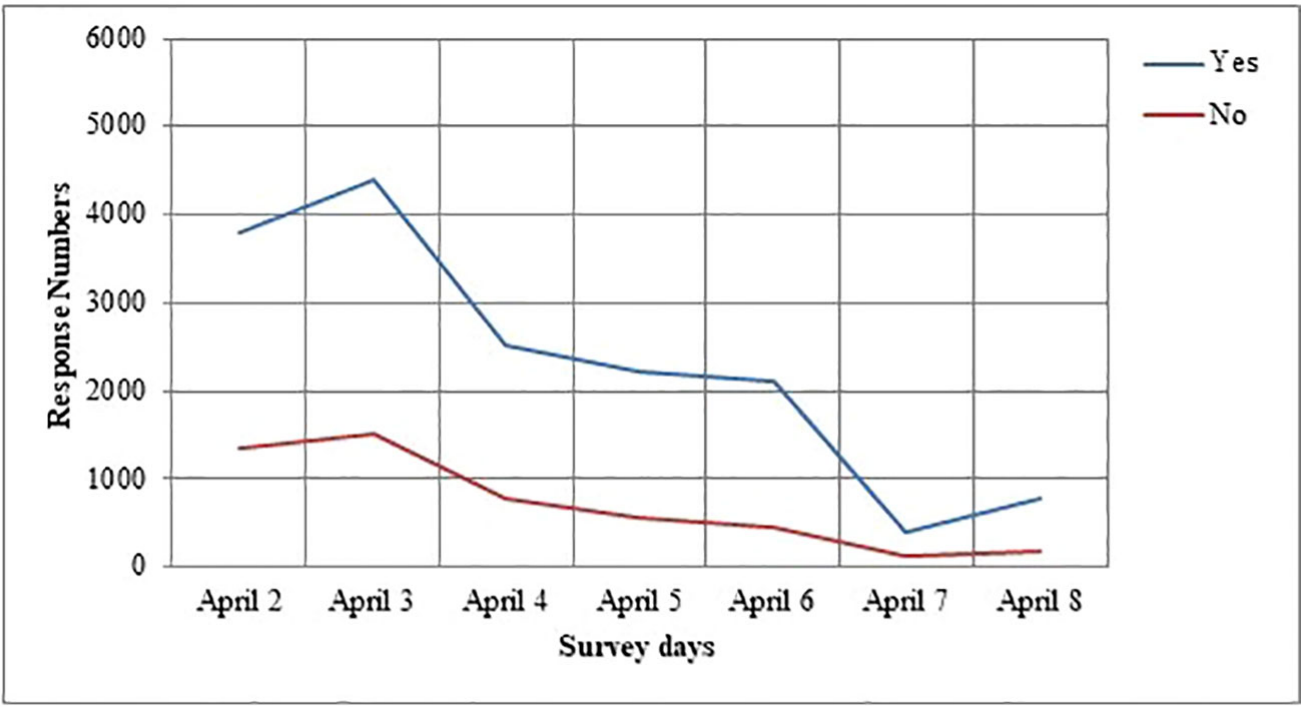
Changes in response numbers of the protection behavior of the survey days.

**Figure 7:**
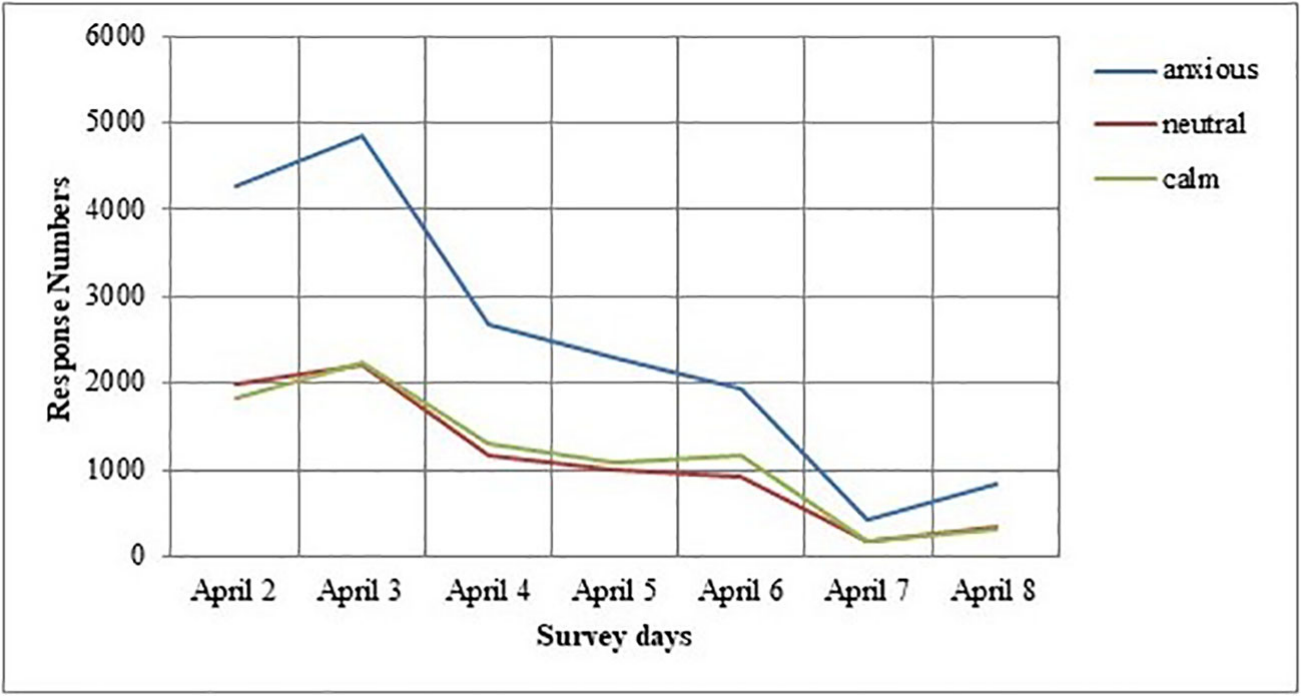
Changes in response numbers of the emotional status of the survey days.

In **Figures 6** and **7**, we plot the change in respondents’ protective behavior and emotional status over the survey days. On the third (April 4th) and sixth day (April 7th) of the study, we see that the number of people reporting a calm emotional state is very high, and the number of people reporting the high values of the protective behaviors is significantly reduced.

So, the current study aimed to determine the prevalence and distribution of anxiety and emotional status and protective behaviors among the young Turkish population and examine their associations with media exposure using a rapid assessment during the COVID-19 outbreak.

## Dıscussıon

Our study is the first study to date that demonstrates the behavioral results of during the COVID-19 outbreak in Turkey. We preferred a web-based survey for assessment of behavioral responses because it is a faster. We found that respondents’ behavior varies regularly with covariates from demographic, epidemiological, media, and emotional status. We determined the protective behaviors and anxiety of people in our country were excessive at the beginning of the survey. Respondents’ fear of their relatives catching the virus was more than the fear they had for catching the virus themselves.

According to the results of our study, among the protective behaviors investigated, social isolation was very high at 97%. As a matter of fact, a study by Filder Smith and Do Freedman also stated that social distances would reduce transmission, as such outbreak diseases require a certain intimacy of people (15). Another surprising point in this study was that use of masks and gloves was higher than we expected because there was no legal obligation to do so. Although some studies (16) emphasize that only individuals with respiratory symptoms should wear a mask, we think that this protection behavior positively reflects the decrease in the number of cases in our country. Another study emphasized that it is very important to wash hands with soap and water before putting on the face mask as well as wearing a face mask (17). In regards to the results we obtained, the handwashing rate of the respondents’ was quite high, and it was higher than the mask-wearing rate.

In response to the question, “What are your emotions about the COVID-19?”, 38% of respondents said “worried”. According to this result, it must be considered normal for respondents to worry about their health. We interpreted this as a positive result that young people have supportive messages and encouraging information. Worry could be increased by misperception in society (18). As evidence from prior outbreaks such as SARS and Ebola showed (19–21) moderate amounts of worrying is effective for controlling the outbreak, but may lead to negative consequences of coronavirus disease control, if it is excessive.

In response to the question, “How scared are you of getting the coronavirus disease (COVID-19)?”, 44% of the respondents stated that they have a moderate level of fear. Strikingly, in response to “How scared are you that a relative will catch the coronavirus disease (COVID-19)?” 80% of respondents said “extremely”. The scare is directly associated with the COVID-19’s rapid and invisible transmission rate, as well as its morbidity and mortality rates (22). It appears that humans perceive it as their moral duty to protect relatives and may exhibit irrational behaviors to do so. Consequently, elevated fears and misconceptions about COVID-19 may result in a disorder of excessive emotional status.

Our study has some public health implications. Our results demonstrate that respondents’ protective behaviors vary consistently with media. Because of strict social isolation precautions, people are maintaining connectivity now more than before using social media and networks, to facilitate human interaction and information sharing about COVID-19. The highest responses to protective behaviors during the COVID-19 outbreak was for social isolation. Previous research shows that respondents did not know that COVID-19 could be transmitted by droplets, which might reduce certain precautionary measures (6). Incompatible with this work were our results showing that respondents use of protective behaviors was high. Effective visual videos, some with famous people, have been shared on social media in our country since the outbreak. Hence it has increased accurate knowledge and positive attitudes of the public about coronavirus disease outbreak. We suggest that providing simple and repeated health education *via* social media is important for encouraging protective behaviors. Our results have revealed that there was a significant correlation between using a computer or mobile phones and sleep patterns. Previous studies support our conclusion that social media, computer games, and the internet cause poor sleep quality (23–27).

When we pay attention to the anxieties caused by COVID-19, the most serious level of responses was fear of losing relatives and future anxiety. Anxiety responses to the feeling of nervousness, sadness, and fear of the worst happening are moderate in our study. The anxiety of the respondents might be result in switching to online education, working from home as much as possible in business life, reducing working hours, social distancing, and other social measures taken across the country. Recent research has indicated that the delay in academic activities was related to the emergence of anxiety symptoms with university students in China (28). Another study has demonstrated that college students’ anxiety about COVID-19 might have been related to the effect of the coronavirus disease on their studies (29) and future employment (6). Prolonged lockdown had several adverse impacts on mental health, especially among young respondents who demonstrated a higher psychological impact of COVID-19 in China (6). Although COVID-19 treatment and vaccine finding studies (30–32) continue around the world, a cure has not yet been found. Consequently, because coronavirus disease does not have an effective treatment, it results in high anxiety responses.

We noted the change throughout the survey in respondent’s protective behavior and emotional status. We observed that respondents’ deployment of protective behavior is affected by their level of the outbreak and current information. We predict that the level of protection and anxious tendencies of people, the adaptation process, and protective behaviors may have been affected by this outbreak. In the study of Jones and Salathe, considering the progress of protection behaviors over time, an increase is observed on the first day, then a sharp decrease, and then a more stable progression is observed (33). Our results showing a linear trend in the perception of outbreak dispersion is associated with a significant decrease in the level of protective behavior and anxiety status in our respondents’ compared to the first survey days. In addition, the behavior and anxiety situation of respondents may be decreased due to the government’s precautions such as the closure of restaurants and intercity restrictions on transportation in our country. In the last of the survey days, we think that the increase in respondents’ anxiety status and protective behavior tendency is a result of the increase in cases in countries such as Italy and Spain. Our research is scientifically important for the study of the spread of knowledge and its relationship to anxiety levels and behavioral change during the most uncertain time of an outbreak.

Several limitations should be noted in the present study. Exposure to news about the COVID-19 outbreak on the internet is not investigated. Meng et al. reported that gender is a biological variable to be considered in the prevention and treatment of COVID-19 (34). In another study, men were emphasized to have worse outcomes and risk of death than women, independent of age, with COVID-19 (35). Considering these studies, an important limitation of this study is that more than half of the participants are female participants. Our participants consisted only of young adults. We did not evaluate the economic status of the participants. Economic vulnerabilities may be the reason for people to seek medical assessment when they present with COVID-19 symptoms (36).

In conclusion, psychological and behavioral researches like this study could help to make progress in building a compassionate person and caring society which would be more effective in preventing and overcoming outbreaks. Our findings to be obtained may shed light on future processes that seem ambiguous for now. Although our study is web-based and has partial limitations for the general population, its rapid implementation, uncovering of unique and critical scientific data may increase the level of public awareness and perhaps lead to life-saving consequences. Public health education programs purposed at improving COVID-19 knowledge can useful encouraging optimistic attitudes towards COVID-19. In addition, cognitive-behavioral therapy can reduce stress and coherent copings (37). University students with good COVID-19 knowledge may reduce negative emotions and deal with the risks from an infection outbreak with a more positive attitude. Our study may have implications for young adult public health provision during outbreaks of infectious disease, including improvements in protective behavior. After the COVID-19 outbreak, studies on the psychological and behavioral effects of the pandemic can also be conducted. The information we have obtained in behavioral dimensions will be an essential scientific reference for other COVID-19 researchers in this vital and critical process and beyond.

## Data Availability Statement

All datasets presented in this study are included in the article.

## Ethics Statement

Written informed consent was obtained from the individual(s) for the publication of any potentially identifiable images or data included in this article. According to the World Health Organization Guidelines on Ethical Issues in Public Health Surveillance, a surveillance study in emergency outbreak situations is clearly exempted from ethical review and oversight (HO guidelines on ethical issues in public health surveillance. Geneva: World Health Organization; 2017. Licence: CC BY-NC-SA 3.0 IGO.). Our online survey was applied in April when the lockdown of Ankara City/Turkey was officially announced. Respondents had to answer a yes-no question to confirm their willingness to participate voluntarily. After confirmation of the question, the participant was directed to complete the self-report survey.

## Author Contributions

GA, MK, and SY conceived and designed the study. SY and MG organized the database. SY, AK, BS, MG, and MK conceived the statistical approach. GA performed the statistical analysis and wrote the manuscript. All authors contributed to the article and approved the submitted version.

## Conflict of Interest

The authors declare that the research was conducted in the absence of any commercial or financial relationships that could be construed as a potential conflict of interest.
